# Saccade Adaptation Abnormalities Implicate Dysfunction of Cerebellar-Dependent Learning Mechanisms in Autism Spectrum Disorders (ASD)

**DOI:** 10.1371/journal.pone.0063709

**Published:** 2013-05-21

**Authors:** Matthew W. Mosconi, Beatriz Luna, Margaret Kay-Stacey, Caralynn V. Nowinski, Leah H. Rubin, Charles Scudder, Nancy Minshew, John A. Sweeney

**Affiliations:** 1 Departments of Psychiatry and Pediatrics, University of Texas Southwestern, Dallas, Texas, United States of America; 2 Departments of Psychiatry and Psychology, University of Pittsburgh School of Medicine, Pittsburgh, Pennsylvania, United States of America; 3 Department of Neurology, Northwestern University Feinberg School of Medicine, Chicago, Illinois, United States of America; 4 Office of the Vice President for Research, University of Illinois, Urbana, Illinois, United States of America; 5 Department of Psychiatry, University of Illinois at Chicago, Chicago, Illinois, United States of America; 6 Departments of Otolaryngology and Neurobiology, University of Pittsburgh School of Medicine, Pittsburgh, Pennsylvania, United States of America; 7 Departments of Psychiatry and Neurology, University of Pittsburgh School of Medicine, Pittsburgh, Pennsylvania, United States of America; University of Reading, United Kingdom

## Abstract

The cerebellar vermis (lobules VI-VII) has been implicated in both postmortem and neuroimaging studies of autism spectrum disorders (ASD). This region maintains the consistent accuracy of saccadic eye movements and plays an especially important role in correcting systematic errors in saccade amplitudes such as those induced by adaptation paradigms. Saccade adaptation paradigms have not yet been used to study ASD. Fifty-six individuals with ASD and 53 age-matched healthy controls performed an intrasaccadic target displacement task known to elicit saccadic adaptation reflected in an amplitude reduction. The rate of amplitude reduction and the variability of saccade amplitude across 180 adaptation trials were examined. Individuals with ASD adapted slower than healthy controls, and demonstrated more variability of their saccade amplitudes across trials prior to, during and after adaptation. Thirty percent of individuals with ASD did not significantly adapt, whereas only 6% of healthy controls failed to adapt. Adaptation rate and amplitude variability impairments were related to performance on a traditional neuropsychological test of manual motor control. The profile of impaired adaptation and reduced consistency of saccade accuracy indicates reduced neural plasticity within learning circuits of the oculomotor vermis that impedes the fine-tuning of motor behavior in ASD. These data provide functional evidence of abnormality in the cerebellar vermis that converges with previous reports of cellular and gross anatomic dysmorphology of this brain region in ASD.

## Introduction

Autism spectrum disorders (ASD) are lifelong neurodevelopmental syndromes that affect social, cognitive, and sensorimotor development [1]. While once considered rare, ASD now are known to affect ∼1 in 88 children, and 1 in 54 males [2]. The etiology(ies) of ASD remains poorly understood. Studies of their neural bases indicate diverse and variably affected brain systems. The cerebellum is one of few brain structures consistently implicated in ASD and in known single-gene disorders associated with ASD [e.g., Fragile X syndrome, Joubert syndrome; [3], [4]]. Reduced Purkinje cell density has been repeatedly documented [5]–[9], and cells in the deep nuclei to which Purkinje cells project are abnormal in size and number [10]. Several studies have indicated that the volume of the cerebellar vermis is smaller in ASD [11]–[16], while cerebellar hemisphere volumes appear to be enlarged [13], [15], [17]–[20] (although see [21], [22] for negative findings).

The cerebellum plays a fundamental role in controlling the precision of movements [23]. Motor abnormalities have been noted in ASD since these disorders were originally described [24], [25]. More recent quantitative studies have documented impaired control of limb movements, including gait alterations, dysmetric manual movements and dyspraxia [26]–[28]. The severity of these abnormalities are predictive of functional outcomes [29] and may be the earliest identifiable features of the disorder [30]–[33]. There is some evidence that the profile of motor control and praxis deficits associated with ASD may be unique to the disorder, but more detailed characterization of motor impairments in ASD is needed [34], [35].

Eye movement studies have shown atypical gaze fixation, increased trial-to-trial amplitude variability of saccadic eye movements, and reduced accuracy of smooth pursuit eye movements in individuals with ASD and their unaffected first-degree relatives [36]–[40]. While initial reports suggested that saccade accuracy is intact in ASD [41], several studies using higher-resolution eye movement monitors have found modest hypometria (i.e., undershooting of targets) [39], [42] that may recover at least partially over the course of development [36]. Functional MRI studies have identified atypical activation in cerebellum, motor cortex, and basal ganglia during tasks of eye and hand movements [43]–[46].

The cerebellum’s role in controlling movement accuracy may be more directly assessed by systematically inducing movement error so that the motor system is forced to adapt [47]–[49]. Adaptation of arm movements involves cerebellum, primary motor cortex and parietal area 5 [50]. Despite the cerebellum’s known role in adapting limb movements, adaptation of limb movements has been shown to be intact in patients with cerebellar ataxia [51]. This suggests that neocortical reorganization may compensate for altered cerebellar adaptation mechanisms. Adaptation of limb movements appears to be unaffected in ASD [52], [53].

Adaptation of saccadic eye movements appears to be primarily dependent on the posterior cerebellum, including lobules VI and VII of the vermis [54]. Selective lesions of the cerebellar vermis lead to long-term disruptions in saccadic eye movement adaptation and the inability to consistently regulate eye movement amplitudes [55], [56]. In non-human primates, hypometria of visually guided saccades also is seen immediately after ablation of the vermis, but appears largely to resolve after only a few weeks [57]–[59]. Saccade adaptation is also altered in patients with cerebellar infarcts, but only if the vermis is affected [56]. It remains to be determined whether saccade adaptation is impaired in ASD.

Saccade adaptation has been well studied using laboratory paradigms in which intrasaccadic shifts in target location are generated immediately after saccade initiation, inducing a relatively constant error in the landing position [60]. Subjects gradually adjust their saccade amplitudes to reduce movement error with practice, although detection of the target displacement is often not consciously recognized because of the visual blanking during saccades.

**Figure 1:**
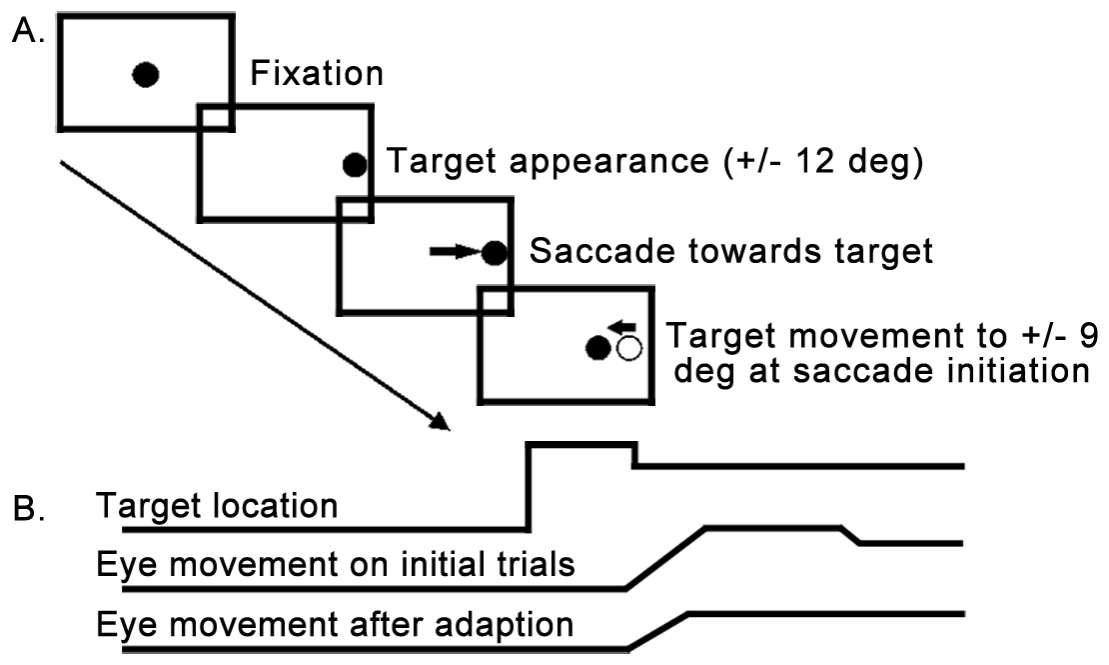
Schematic representations of the saccade adaptation test. A.) Participants were instructed to shift their gaze to peripheral targets when they appeared. After subjects initiated their saccade, the target stepped inward from the ±12 deg location to ±9 deg in the same visual hemifield. B) Schematic representations of double-step target displacement and traces of non-adapted and adapted saccades.

In the present study, we utilized a conventional intrasaccadic target step paradigm to study saccade adaptation and the integrity of the cerebellar vermis in individuals with ASD (Figure 1). We reasoned that if individuals with ASD have a deficit involving the cerebellar vermis, then they will adapt at a slower rate than healthy controls, and they will show greater trial-to-trial variability in the amplitude of their saccades. We also predicted that cerebellar-dependent motor deficits evident in the oculomotor system would be associated with motor control impairments in the manual system. Therefore, we examined saccade adaptation rates and amplitude variability in relation to performance on a traditional neuropsychological test of manual motor control.

## Materials and Methods

### Subjects

Fifty-six individuals with ASD (50 M, 6 F) and 53 healthy control individuals (46 M, 7 F) participated in this study (Table 1). Due to the large number of trials required for subjects to adapt and then recover, subjects were randomly assigned to have either leftward or rightward adaptation tested (346 total trials, including 50 baseline trials of visually guided saccades, 200 trials during adaptation testing, and 96 trials of post-adaptation recovery). Thus four groups were examined (ASD leftward: n = 29; ASD rightward: n = 27; control leftward: n = 23; control rightward: n = 30). No demographic or performance differences between leftward and rightward adapting groups were identified, so they were combined for analyses. The ASD and control groups were matched on age (range 8–54 years) and handedness. Subjects completed the Wechsler Abbreviated Scale of Intelligence to assess current intellectual abilities [61]. The subject groups had similar Performance IQs, but the ASD group had significantly lower Verbal and Full-Scale IQs than the control group. All IQ scores were in the average range for both groups.

**Table 1:** Demographic characteristics of individuals with autism spectrum disorders (ASD) and healthy control subjects.

	ASD		Controls		
	(n = 56)		(n = 53)	t	p
Age		19 (9)	18 (8)	.16	.87
% Male^1^		89	87	.16	.69
Verbal IQ		101 (14)	108 (11)	*3.28*	*<.01*
Performance IQ		105 (16)	108 (9)	1.87	.07
Full Scale IQ		103 (15)	110 (10)	*2.97*	*<.01*
Manual Motor Timeto Completion (s)		97.99 (29.74)	74.43 (9.20)	*4.65*	<.01
Manual Motor Errors		1.05 (1.18)	.68 (.69)	1.72	.09

Values are Mean (SD).

IQ = Intelligence quotient; s = seconds.

1 Chi-Square statistic.

Individuals with ASD, including Autistic Disorder and Asperger Disorder, were recruited through community advertisement at the University of Pittsburgh. Diagnoses of ASD were established with the Autism Diagnostic Inventory-Revised (ADI-R; [62]) and the Autism Diagnostic Observation Schedule (ADOS; [63]) and confirmed by expert clinical opinion. Participants with ASD were excluded if they had a known genetic or metabolic disorder associated with ASD (e.g., Fragile X syndrome, tuberous sclerosis).

Control participants were recruited from the community through newspaper advertisements. Potential control participants completed a screening questionnaire that included prenatal, birth and developmental history, general medical history, treatment/medication history, and personal and family history of psychiatric and neurological disorders. Parents of controls less than 18 years old completed these questionnaires for their children. Control subjects were excluded for current or past psychiatric or neurological disorders, family history of ASD in first-, second- or third-degree relatives, or a history in first-degree relatives of a developmental or learning disorder, psychosis, or obsessive compulsive disorder.

No subjects were taking medications known to affect eye movements at the time of testing, including antipsychotics, stimulants or anticonvulsants [64]. Subjects had corrected or uncorrected far visual acuity of at least 20/40. No participant had a history of head injury, birth injury or seizure disorder. After a complete description of the study, written informed consent was obtained for each adult participant and informed parental consent was obtained for individuals less than 18 years of age. Minors provided written assent. Study procedures were approved by the Institutional Review Board of the University of Pittsburgh.

### Eye Movement Tasks and Procedures

Saccade adaptation was elicited using an intrasaccadic target displacement paradigm with a centripetal step. Circular targets subtending 0.5 degrees (deg) of visual angle were presented in the horizontal plane at eye level on a computer monitor with a 57 cm wide screen display (Sony PREMIERPRO Series 24 FD Trinitron® CRT, Model GDM-FW900). Spatial resolution was set at 2304×1440 pixels. Subjects were seated 69 cm from the monitor and tested alone in an unlit room with flat black walls. The horizontal display subtended 23 deg of visual angle (29 cm) to each side of center. A chin rest with forehead and occipital restraints was used to minimize head movement.

Eye movements were monitored using infrared reflection sensors mounted on spectacle frames (Applied Science Laboratories, Inc., Model 210). Blinks were identified using electrodes placed above and below the left eye linked to an AC-coupled bioamplifier. Prior to performing tasks, subjects fixated a central target and peripheral targets at +/−12 deg of visual angle to calibrate eye movement recordings. An examiner in an adjacent room monitored eye movement activity during testing to ensure that subjects were alert and performing tasks according to instructions. Subjects performed the following three tasks:

#### Baseline saccade task

To measure saccade amplitudes prior to adaptation, subjects first performed 50 trials of visually guided saccades. Trials began with central fixation for 1000–1500 ms, after which the target stepped to +/−6, 12 or 16 deg in the horizontal plane. Fifty percent of the trials were presented at the location from which saccades were to be adapted during the adaptation task (i.e., +12 deg for rightward adapting subjects, or −12 deg for leftward adapting subjects). Target presentation in the remaining trials was equally distributed across the other five locations.

#### Saccade Adaptation Task (Figure 1)

Trials began with central target fixation for 1000–1500 ms. Subjects were required to maintain fixation within +/−3 deg of center for at least 500 ms immediately before peripheral targets appeared. Adaptation trials constituted 90% (180/200) of trials during this phase of testing. During adaptation trials, the target stepped 12 deg from central fixation. When saccade velocity exceeded 30 deg/s, close to saccade initiation, targets were centripetally displaced from 12 to 9 deg from central fixation. The target remained at 9 deg for 1000 ms in 90% of adaptation trials. It remained at that location for 2000 ms in the remaining 10% of adaptation trials to encourage sustained fixation of the 9 deg target rather than an immediate return to central fixation. In 20 randomly interspersed trials (10% of adaptation trials), targets were presented at 6 deg from center in the hemifield opposite that in which adaptation was tested. These “catch trials” were presented without an intrasaccadic displacement, and were intended to reduce rates of anticipatory saccades to the +/−12 deg location. In this type of saccade adaptation paradigm, subjects gradually learn to make saccades closer to the 9 deg location, rather than to the 12 deg location. Typically, because the 3 degree centripetal step occurs during a saccade, the displacement is not perceived by subjects. Two hundred trials were presented in four 50-trial blocks, with 15 s of darkness between blocks to allow subjects to rest.

#### Recovery task

To evaluate recovery from adaptation, subjects completed 80 trials (83% of recovery trials) in which they made saccades to stimuli presented 12 deg from fixation in the same hemifield in which adaptation had been tested. These trials did not include any intrasaccadic target displacement, so that recovery from adaptation effects could be examined. This task also included 16 “catch trials” (17% of post-adaptation trials) in which targets were presented at 6 deg in the opposite hemifield.

### Eye Movement Measurements

Eye movement signals were sampled at 500 Hz with a 14-bit A/D converter (Dataq Instruments, DI-210). Recordings were smoothed off-line with a finite impulse response filter. The filter had a gradual transition band (from pass to no pass) between 20 Hz and 65 Hz for velocity and position data, and 30 Hz and 65 Hz for acceleration data. Saccades were identified for scoring when eye acceleration exceeded 1000 deg/s^2^, and were measured until 25% of peak deceleration, permitting resolution of saccade detection on the order of 0.25 deg. Recordings from the right eye were scored, unless there were problems with this recording (i.e., signal clipping or high noise artifact). Data were scored without knowledge of subjects’ diagnosis or demographic characteristics.

### Manual Motor Testing

Participants completed a manual motor test in which they were presented with a pegboard of keyholes arranged in a 5×5 grid and with various orientations (Lafayette Instruments, Lafayette, IN). Subjects were instructed to insert a grooved peg into each slot with the proper orientation as fast as possible. Their dominant and non-dominant hands were tested separately. Time to completion and the number of times a peg was dropped were examined.

### Statistical Analyses

Because amplitudes did not change over time during baseline testing, each subject’s amplitude values were averaged over all 12 deg trials in the direction to which adaptation was tested. To examine the rate of adaptation, we used general linear mixed models because this type of analysis is robust to missing data and it increases the precision of slope estimates by providing estimates for each individual [65]. In the present study, the logarithmic transformation of trial number was entered as a repeated measurement (to accommodate nonlinear learning functions), diagnostic group (ASD, controls) and the group X trial number interaction terms were entered as independent variables, and saccade amplitude was the dependent variable. These models yielded separate estimates of learning rate for each group as well as estimates of group differences. The same approach was used to evaluate amplitude change during the recovery phase, but a linear model is presented as it provided a good fit to the data. To examine the trial-wise variability of saccade amplitude, trials with target locations to which subjects adapted were divided into blocks of 10 sequential trials for baseline, adaptation and recovery phases. Then, the standard deviation of saccade amplitude was computed to assess amplitude variability for each block for each individual. This yielded 3 blocks for baseline data (the third block consisted of only 5 trials), 18 blocks of adaptation trials and 8 blocks of recovery trials. Linear (for baseline testing) and logarithmic models (for adaptation and recovery testing) were used to examine changes in amplitude variability during baseline, adaptation and recovery separately, but subjects did not show changes in amplitude variability within any phase (baseline: β = –.01; SE_1,319_ = .03×10^−1^; Z = −2.01; p = .05; adaptation: β = .24×10^−3^; SE_1,1954_ = .34×10^−3^; Z = .71; p = .48; recovery: β = .42×10^−2^; SE_1,1082_ = .31×10^−2^; Z = 1.36; p = .18). Therefore, blocks were averaged within each phase for analyses.

For all tasks, trials were considered invalid and excluded from analyses if primary saccades occurred simultaneously with a blink, were made in the wrong direction, or were anticipatory (latency <70 ms). Saccades more than 3 SD outside of the mean amplitude for all subjects were excluded from analyses to reduce outlier effects. For these reasons, the number of trials analyzed per individual with ASD on the adaptation task was less than that for controls (ASD: 142 (20); controls: 154 (16); t_107_ = 3.24, p = .001), but the number of trials for each group was sufficient to analyze learning rates. Further, the number of trials analyzed per subject was not related to adaptation rate (r_108_ = –.13, p = .18). Analyses of adaptation rate and amplitude variability each were performed with and without age entered as a covariate. Including age as a covariate did not substantively impact the results, so it was not included in the final models.

## Results

### Saccade Amplitude

**Figure 2:**
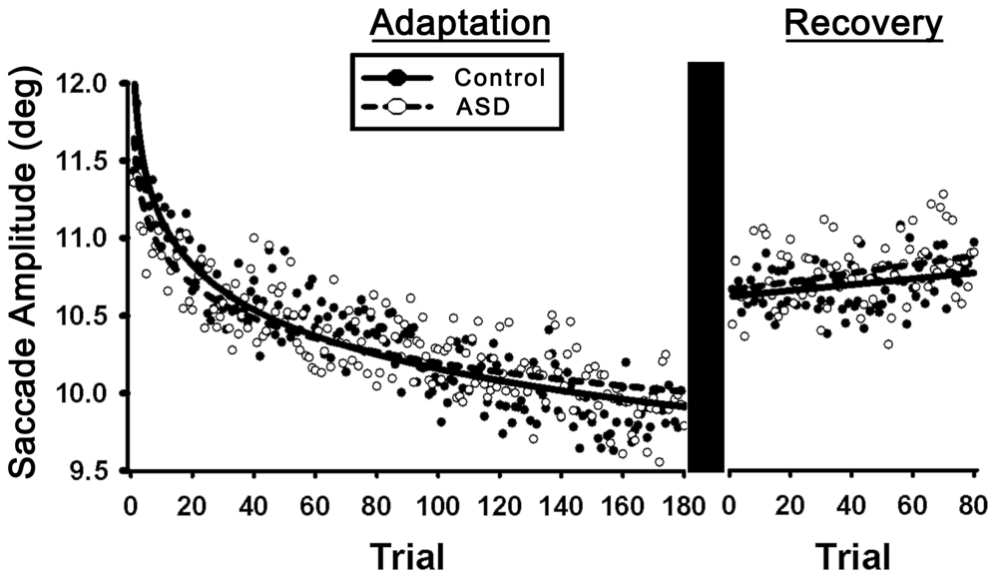
Saccade amplitudes for subjects with autism spectrum disorders (ASD) and healthy controls during adaptation and recovery. The natural logarithmic model fit is presented for adaptation, and each data point represents the group mean for an individual trial. Subjects with ASD showed reduced rates of learning (p<.01). The linear fit is presented for recovery data. There was no difference in the rate at which subjects with ASD and healthy controls increased their amplitudes during the recovery phase (p = .66).

During the baseline condition, subjects with ASD and healthy controls did not differ in mean saccade amplitude (t_107_ = 1.23, p = .22; Table 2). During adaptation, healthy subjects (β = –.41; SE_1,15902_ = .02; Z = −17.51; p<.01) and subjects with ASD significantly reduced their saccade amplitudes over trials (β = –.31; SE_1,15902_ = .03; Z = 10.61; p<.01). Subjects with ASD adapted at a slower rate than controls (Figure 2; β = .11; SE_1,15902_ = .04; Z = 2.83; p<.01).

**Table 2:** Saccade amplitude and amplitude variability on 12 deg trials for individuals with autism spectrum disorders (ASD) and healthy controls during baseline testing, adaptation, and post-adaptation recovery.

	ASD	Controls	t	p
**Baseline**				
Mean Amplitude (deg)	11.75 (.58)	11.79 (.45)	1.23	.22
Amplitude variability (deg)	0.84 (.40)	0.69 (.33)	*2.13*	.*03*
**Adaptation**				
Rate of amplitude change^a^	β = -.31 (SE = .03)	β = –.41 (SE = .02)	*2.83* b	*<.01*
Amplitude variability (deg)	1.45 (.47)	1.17 (.38)	*3.37*	*<.01*
**Recovery**				
Rate of amplitude change^a^	β = .02×10^−1^ (SE = .05×10^−2^)	β = .02×10^−1^ (SE = .02×10^−1^)	.04^b^	.97
Amplitude variability (deg)	1.10 (.40)	0.89 (.39)	*2.74*	*<.01*

Values are Mean (SD).

a negative values indicate reduction over trials.

b z-score for comparisons of slopes between subjects with ASD and controls.

The rate at which subjects adapted was greatest during initial trials of adaptation; specifically, subjects achieved 66% of their total amplitude reduction by trial 30. Therefore, we tested whether subjects with ASD also showed adaptation deficits during this initial phase of rapid amplitude reduction. ASD subjects adapted slower during the first 30 trials than healthy controls (β = .13; SE_1,2743_ = .06; Z = 2.01; p = .04).

**Figure 3:**
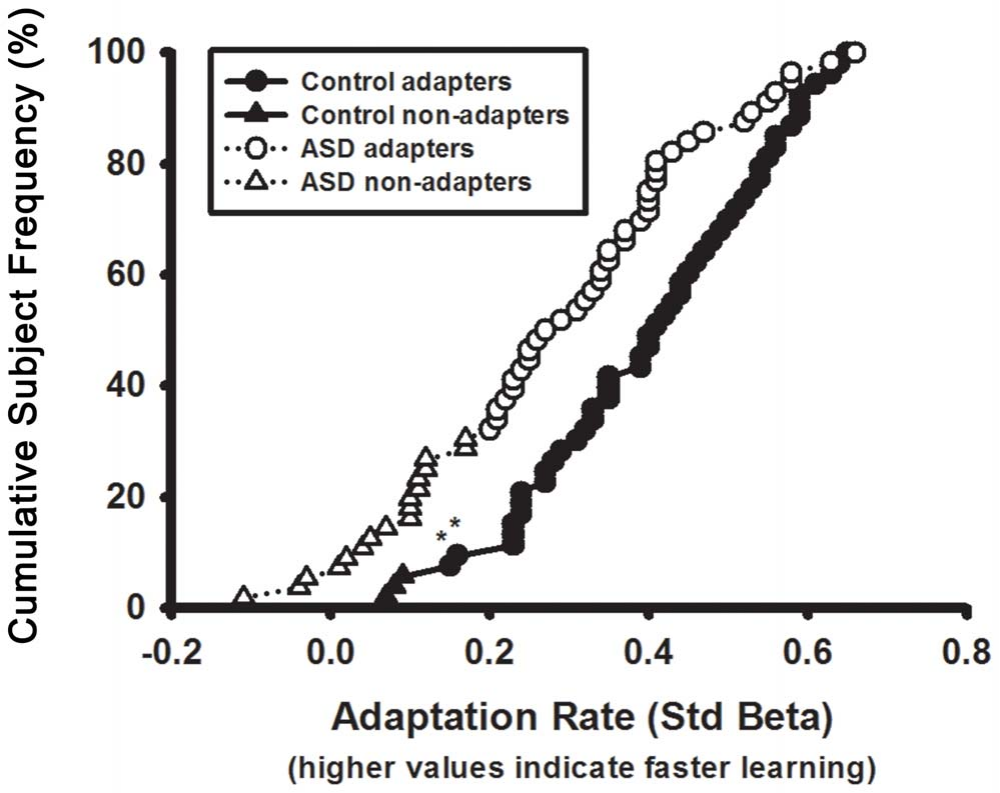
Cumulative frequency of adaptation rates (i.e., rate of amplitude reduction) for subjects with autism spectrum disorders (ASD) and healthy controls. Positive values represent faster learning rates. Triangles are used to represent non-adapters for both diagnostic groups. Asterisks (*) are used to identify two control subjects whose rates of adaptation were statistically significant (p<.05) but also less than those of two non-adapting subjects with ASD. These two controls made more saccades that could be scored during adaptation (151 and 170 out of 180 respectively) than the two non-adapting subjects with ASD whose learning rate was faster but non-significant (133 and 135 out of 180 respectively). If these two subjects with ASD are re-classified as adapters, there is still a higher proportion of non-adapting ASD subjects compared to healthy controls (ASD: 27% (15/56); controls: 6% (3/53); X^2^_1_ = 8.81, p<.01).

We compared the proportion of individuals with ASD and healthy controls who did not adapt by classifying subjects whose rate of change over adaptation trials was not significant (p>.05) as “non-adapters”. A higher proportion of ASD subjects were non-adapters compared to healthy controls (Figure 3; ASD: 30% (17/56); controls: 6% (3/53); Χ^2^ = 11.09, p<.01). There were no differences between subjects with ASD that adapted versus those that did not adapt in terms of age (t_53_ = .10, p = .93), IQ (t_53_ = 1.12, p = .24), ADI-R social scores (t_53_ = .29, p = .77), ADI-R communication scores (t_53_ = 1.06, p = .29), ADOS social-communication scores (t_53_ = .24, p = .81), or baseline saccade amplitudes (t_53_ = .91, p = .37).

A comparison of mean saccade amplitudes during recovery indicated that they remained decreased relative to baseline saccade amplitudes in both groups (Figure 2; F_1,104_ = 248.42, p<.01), which is typical for this type of paradigm and number of recovery trials [66], [67]. Overall, subjects showed a linear increase in their saccade amplitudes during recovery trials (β = .03×10^−1^; SE_1,7357_ = .01×10^−1^; Z = 2.18; p = .03). The rate of recovery did not differ between subject groups (β = .03×10^−3^; SE_1,7357_ = .07×10^−2^; Z = .04; p = .97).

### Amplitude Variability across Trials

**Figure 4:**
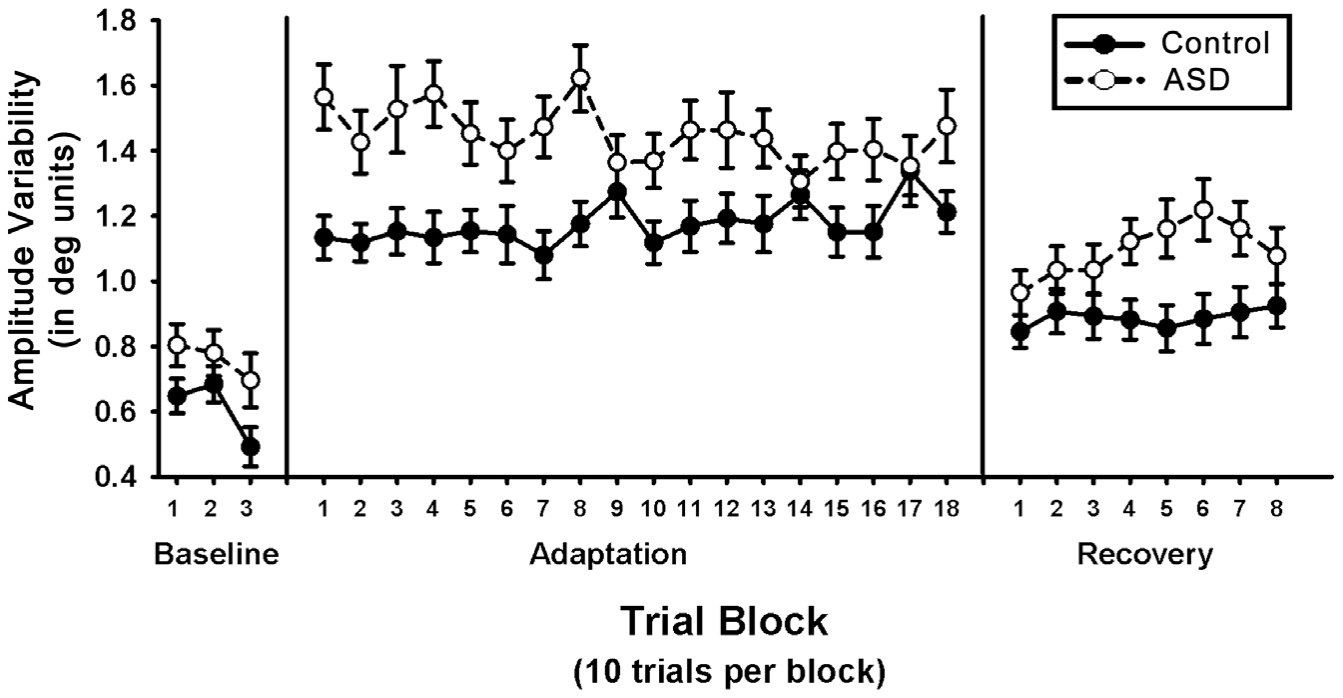
Standard deviation of the amplitude of primary saccades, a measure of trial-wise variability in saccade accuracy, during baseline testing, adaptation and recovery. Data are presented in blocks of 10 trials. Subjects with autism spectrum disorders (ASD) showed greater trial-wise variability in saccade accuracy during each phase of testing (p<.01), but the rates at which variability changed during each of the phases were not different between subjects with ASD and healthy controls (p>.10).

Subjects with ASD showed more trial-wise variability in saccade amplitudes relative to controls across conditions (baseline, adaptation, and recovery; F_1,106_ = 11.32, p<.01). We analyzed each condition separately and found that subjects with ASD showed increased trial-wise amplitude variability prior to adaptation (Table 2; t_107_ = 2.13, p = .03), although this difference was no longer significant when a Bonferroni correction for multiple comparisons was applied (p<.017). Across groups, amplitude variability was greater during adaptation trials compared to baseline trials (F_1,105_ = 141.27, p<.01). During adaptation, subjects with ASD continued to show greater mean amplitude variability than controls (Figure 4; t_107_ = 3.37, p<.01), but the degree to which amplitude variability increased during adaptation relative to baseline did not differ between subjects with ASD and controls (mean (sd) % increase: ASD = 82% (81); controls = 89% (96); t_107_ = .43, p = .67). During the recovery phase, individuals with ASD continued to show more amplitude variability than controls (t_107_ = 2.74; p<.01). Non-adapting subjects with ASD showed greater amplitude variability than adapters with ASD during adaptation (t_54_ = 3.68, p<.01) and during recovery (t_54_ = 2.93, p<.01), but not at baseline (t_54_ = 1.17, p = .25).

### Correlational Analyses

**Figure 5:**
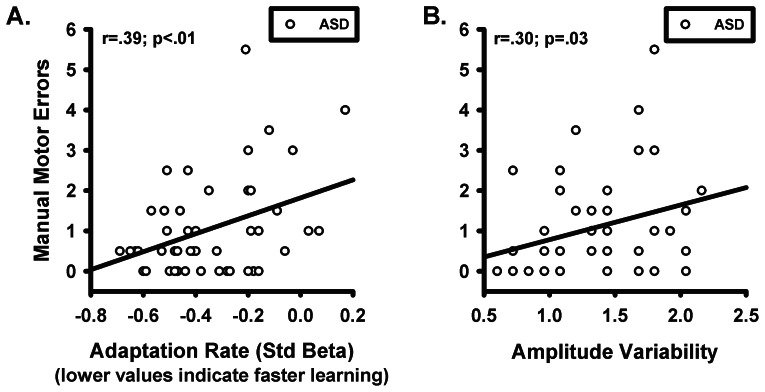
Relationships between saccade adaptation and manual motor performance. The number of errors during the manual motor test were averaged across hands and are presented for subjects with ASD in relation to A) the rate at which they adapted and B) their trial-to-trial amplitude variability across baseline testing and the adaptation and recovery phases.

Neither the rate of adaptation nor amplitude variability was associated with age or IQ (for either group), or social-communication impairments on the ADOS or ADI-R for ASD participants (see Tables S1 and S2). Subjects with ASD were slower to complete the manual motor test (F_1,86_ = 21.65, p<.01), but there was no difference between groups in the number of dropped pegs (F_1,86_ = 2.96, p = .09). The group x hand (dominant vs. non-dominant) interactions were not significant (time to completion: F_1,75_ = .11, p = .74; dropped pegs: F_1,75_ = .08, p = .78), so performance was averaged across hands for correlational analyses. We used a Bonferroni corrected alpha level <.013 for these analyses. For subjects with ASD, adaptation rate was not associated with manual motor time to completion (r_56_ = .11, p = .43), but slower learning rates were associated with more dropped pegs during manual testing (Figure 5A; r_56_ = .39, p<.01). Neither of these relationships were significant for healthy controls (time to completion: r_37_ = .22, p = .19; dropped pegs: r_37_ = .18, p = .30). Increased amplitude variability in subjects with ASD showed a modest non-significant relationship with the number of dropped pegs, (Figure 5B; r_40_ = .30, p = .03). Amplitude variability was not related to manual motor completion time in subjects with ASD (r_56_ = -.04, p = .79). The relationships between saccade amplitude variability and manual motor indices were not significant for healthy controls (time to completion: r_37_ = .21, p = .22; dropped pegs: r_37_ = .17, p = .31). Increased amplitude variability during adaptation was related to reduced rates of adaptation for both healthy controls (r_53_ = .50, p<.01) and subjects with ASD (r_54_ = .58, p<.01). For both groups, less amplitude variability predicted faster adaptation.

### Performance On 6 Degree “Catch Trials” During Adaptation And Recovery

Subjects showed a reduction in saccade amplitudes on the catch trials during adaptation relative to baseline saccade performance (F_1,107_ = 24.36, p<.01). However, the level to which amplitudes were reduced (4%) was less than that seen for adaptation trials with an intrasaccadic displacement (19%; F_1,107_ = 233.56, p<.01) and is less than the range of amplitude reduction for adapted saccades (10–40%) reported in previous studies [67]–[69]. The degree to which amplitudes were reduced on adaptation catch trials relative to saccades to the same location during baseline testing did not differ between individuals with ASD and healthy controls (F_1,107_ = .29, p = .59). Saccade amplitudes during recovery catch trials were greater than adaptation catch trials (F_1,106_ = 4.19, p = .04) but did not differ between subject groups (F_1,106_ = .02, p = .88). Variability in saccade amplitude for 6 deg trials also increased during adaptation compared to baseline trials (F_1,105_ = 46.21, p<.01), but the degree to which it changed did not differ between groups (F_1,107_ = .31, p = .58). No changes in amplitude variability occurred from adaptation to recovery for catch trials (F_1,106_ = .65, p = .42).

## Discussion

We used a saccadic adaptation test to examine how individuals with ASD adjusted their eye movements to systematically induced retinal image position errors [60]. This test requires that individuals refine their movement trajectories over trials to minimize error in the focus of gaze at the completion of saccades. Saccade adaptations are thought to be organized within the oculomotor vermis [54],[70], a region implicated in post-mortem and MRI studies of ASD [6],[10],[71]. In the present study, individuals with ASD adapted at a slower rate than healthy controls, suggesting that learning mechanisms within the oculomotor vermis are compromised. Our finding that saccade adaptation was altered despite no impairment being observed in the average accuracy of visually guided saccades during baseline testing indicates that cerebellar circuitry supporting on-line refinements of eye movements may be selectively disrupted in ASD. Alternatively, compensatory processes in other brain regions may be sufficient to minimize errors in gaze shifts over a longer period of time (days or weeks), so that the accuracy of visually guided saccades in individuals with ASD is less affected [36],[39].

The amplitude variability reducing function of the cerebellum also was abnormal in individuals with ASD. Increased trial-to-trial variability of saccade amplitudes was associated with reduced rates of adaptation, consistent with the hypothesis that vermal dysfunction contributes to both impaired adaptation and a failure to compensate for within-trial variability within the saccadic system, such as in the cumulative neuronal activity in the superior colliculus before and during the saccade. It also is possible that the increased variability of saccade landing positions, and thus the reduced consistency of the error signal relayed to the cerebellum reduces the rate at which adaptive mechanisms within the vermis can make systematic adjustments to saccade trajectories [72]. Studies in which the degree of error is systematically controlled for each individual subject are needed to address the relative contribution of increased amplitude variability to adaptation impairments in ASD.

Our results confirm and extend previous reports describing saccadic eye movement deficits in ASD. Prior studies documenting modest hypometria and greater variability in amplitude gain provide indirect evidence that cerebellar control of saccade accuracy may be compromised [36],[39]. Functional MRI findings of reduced vermal activation during saccades in ASD provides additional support for cerebellar alterations during eye movements, but the extent to which these alterations are a consequence of or causes to dysfunction within frontal and parietal cortical eye fields and basal ganglia nuclei remains unclear [46]. The present findings show that brain circuitry supporting the rapid adjustment of saccadic amplitudes to reduce retinal error is abnormal in ASD. There is considerable evidence from non-human primate work and studies of human cerebellar patients (described below) that modifications of Purkinje cell output within the vermis are primarily responsible for the short-term adaptation of saccades. Therefore, this study provides evidence, perhaps as strong as any available neurobehavioral paradigm can provide, that the function of the cerebellar vermis is disrupted in ASD and its dysfunction may underpin the motor coordination and learning impairments characteristic of this disorder.

The pattern of increased trial-to-trial variation in saccade accuracy together with a lack of alteration in average saccade accuracy during baseline testing is similar to that seen after monkeys have recovered from lesions in the oculomotor vermis [73]. Acute effects include dysmetria, more variable amplitudes, and reduced or abolished short-term adaptation. The ability to make accurate saccades is recovered over time, whereas amplitude variability and adaptation deficits persist. Thus, while brain regions outside of the vermis may be able to compensate for smaller amplitude errors over extended periods of time during saccades to static targets, they cannot sufficiently support rapid, larger scale corrections such as those elicited by adaptation paradigms. Instead, these larger scale corrections are uniquely dependent on the vermis [74].

A pattern similar to that seen in chronic lesions may occur in a neurodevelopmental disorder such as ASD, with putative alterations of the cerebellar vermis occurring within the first years of life [7]. It is possible that brain systems controlling the accuracy of saccades may be rescued at least partially during development, a hypothesis consistent with findings from a study of ASD documenting saccadic hypometria in children, but not adolescents or adults with ASD [36]. Adaptation mechanisms do not appear to recover, as we did not observe age-related differences in adaptation rate across the relatively wide age range of our study sample. Still, some caution should be exercised in interpreting the absence of developmental group differences as longitudinal studies would be necessary to characterize the maturation of adaptive mechanisms in ASD. Notably, baseline testing in the present study was designed specifically to assess saccade amplitudes to a particular location from which adaptation would be measured. The increased predictability of target locations (50% of trials were to the to-be-adapted location) may have limited our ability to identify the modest differences in saccade amplitude gain that have been reported in some prior studies [36],[39]. Similarly, no amplitude differences were seen between groups during “catch trials”, suggesting that there may have been too few trials to detect deficits, or that the accuracy of smaller saccades is relatively preserved in ASD as we have reported previously [37],[41].

### The Neural Circuitry Of Adaptation Deficits In Asd

**Figure 6:**
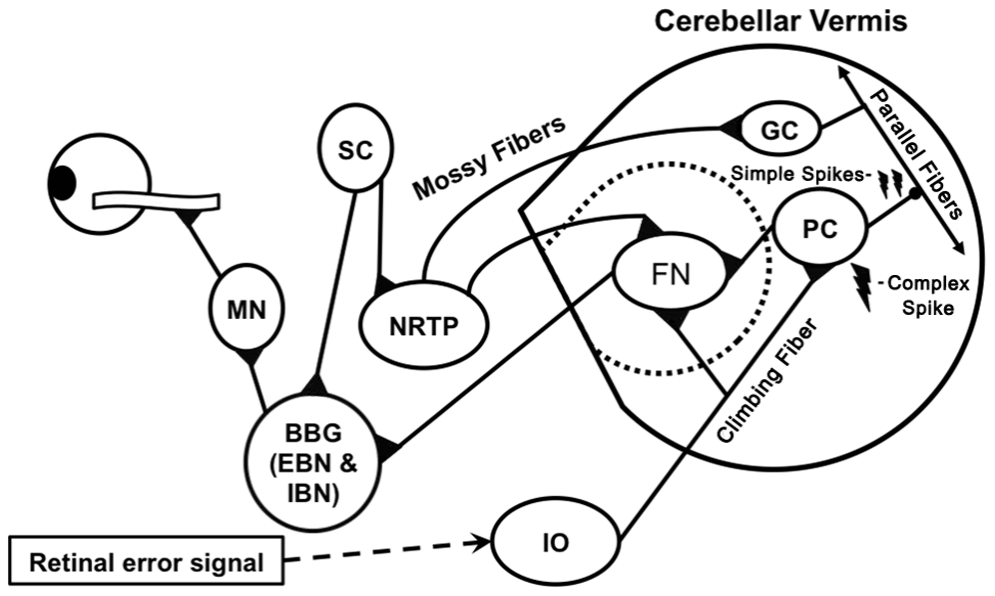
Figure 6. Schematic representation of a sagittal view of the cerebellar “oculomotor” vermis - brainstem circuitry involved in the adaptation of saccadic eye movements. Retinal error information reaches the inferior olive (IO), which in turn projects ascending climbing fibers that create complex spike action potentials at synapses on the Purkinje cell (PC) body and proximal dendrites. PCs also receive input via mossy fibers projecting from the nucleus reticularis tegmenti pontis (NRTP), which receives a saccade command from the superior colliculus (SC). Rapid, simple spike discharges occur at the synapses of mossy fiber-parallel fiber inputs to PCs. The complex spike firing of the climbing fiber-PC synapses induces a long-term depression (LTD) of simple spikes that is thought to be the mechanism guiding saccade adaptation. Changes in PC simple spike bursts modify saccade trajectories by altering the level of inhibitory output to fastigial nuclei (FN), which in turn modulate inhibitory (IBN) and excitatory burst neuron activity (EBN) in the brainstem burst generator (BBG). The BBG innervates abducens motoneurons (MN) that control horizontal eye movements via lateral and medial extraocular muscles.

The cerebellum supports motor adaptation by monitoring the difference between expected and observed movement outcomes, adjusting movements on-line and refining future motor commands [75],[76]. The oculomotor vermis receives retinal error signals via olivary climbing fibers (Figure 6) [77]–[79]. Olivary inputs create complex spike discharges which, when co-occurring with more frequent simple spike discharges at parallel fiber-Purkinje cell synapses, can lead to a long lasting depression (LTD) and selective pruning of the parallel fiber input [80]. These processes modulate the amplitude and timing of Purkinje cell population bursts converging on fastigial neurons, and are the plastic mechanisms thought to support saccade adaptation [81]. Inactivation of vermal Purkinje cells eliminates short-term saccade adaptation and produces more variable saccades across trials in monkeys [59],[82]. Patients with cerebellar disease show saccade adaptation deficits and an impaired ability to modulate saccade accuracy across trials only if the vermis is affected [56]. The similarity between the pattern of deficit seen in patients with vermal lesions and individuals with ASD suggests that alterations in the oculomotor vermis are the primary factor responsible for saccade abnormalities observed in the present study.

The majority of post-mortem brain studies of ASD examining the cerebellum have documented cellular and neurochemical alterations [6],[7],[9],[83]–[86]. Still, some individuals examined in post-mortem studies were taking anticonvulsant medications that could selectively affect Purkinje cell neurochemistry and anatomy. Adaptation abnormalities in medication-free individuals with ASD observed in the present study indicate that functional alterations implicating the vermis are not a result of medication effects.

A relatively high number of ASD subjects (30%) did not significantly adapt. This suggests variability in the extent to which the cerebellar vermis is functionally impaired in individuals with ASD, and these results might indicate the presence of a subgroup of individuals with more severe oculomotor disturbances. However, there was no clear bimodality in the distribution of adaptation rate, nor did we identify clinically distinct features of subjects who did not adapt. Further phenotypic and genotypic characterization of non-adapters and their unaffected family members may help resolve the possibility that they represent a distinct subgroup of individuals with ASD.

Manual motor error rates (dropping pegs during a pegboard test) were associated with saccadic adaptation rates in individuals with ASD, suggesting that cerebellar dysfunctions may involve circuits supporting motor control across multiple effectors. The clinical neuropsychology measures of manual motor function did not detect deficits in manual control or speed in subjects with ASD. This suggests that oculomotor studies may provide a more sensitive methodology for identifying impairments or that abnormalities are more profound in the oculomotor system. However, it is important to note that the measure of manual motor control used in the present study is likely not sensitive to subtle dysmetria or altered kinematics of movement. Direct comparisons of oculomotor and manual motor control utilizing similarly sensitive neurophysiological measures are needed to contrast eye and limb movement control in ASD.

We cannot completely rule out the possibility that adaptation deficits seen in individuals with ASD also may reflect dysfunctions in other brain regions. It has been suggested that retinal error signals may originate in the superior colliculus [87], although recent evidence indicates that motor activity in the superior colliculus is unaltered during saccade adaptation [88]. Inactivation of the superior colliculus typically affects non-adapted saccades as well, altering trajectories, lowering peak velocities, and extending durations and latencies [89]. Neither the present study (Table S3) nor previous reports examining non-adapted saccades suggest that saccade dynamics are disrupted in ASD [36],[39],[41] (although see [39] for evidence of reduced peak velocity of visually guided saccades in ASD).

Lesions affecting ventral and lateral thalamic nuclei impair saccade adaptation [90], suggesting that adaptation deficits in ASD could reflect alterations in these nuclei. Frontal and parietal eye fields also are involved in the control of saccade amplitudes, although there is less evidence that they are involved in adaptation of exogenously driven saccades such as those studied here [54]. Still, while there is a relatively strong rationale for interpreting the present neurophysiological findings as providing evidence for vermal dysfunction in ASD, research is needed to further link these behavioral observations to direct physiological studies of the vermis.

### Conclusions

There is considerable evidence of cellular and gross anatomic changes of the vermis in ASD [10],[91]. The present study offers perhaps the most direct evidence to date that these alterations have functional implications in this disorder. Our study shows that individuals with ASD are not able to rapidly adjust to large errors in saccade landing position. They also have poor control over the consistency of their movement amplitudes as evidenced by greater trial-to-trial variability. We hypothesize that important plastic mechanisms within the cerebellar vermis involving Purkinje cell output to fastigial nuclei are impaired in ASD. The failure of these learning mechanisms may contribute to the motor control impairments in ASD and may have direct implications for understanding the cellular and molecular bases of this disorder.

## Supporting Information

Table S1 **Relationships between adaptation performance and clinical/demographic characteristics for subjects with Autism Spectrum Disorder (ASD).**
(DOC)

Table S2 **Relationships between adaptation performance and demographic characteristics for healthy control subjects.**
(DOC)

Table S3 **Saccade characteristics of individuals with autism spectrum disorders (ASD) and healthy controls during baseline testing, adaptation and recovery.**
(DOC)
