# Supplementary material for: Saccade Adaptation Abnormalities Implicate Dysfunction of Cerebellar-Dependent Learning Mechanisms in Autism Spectrum Disorders (ASD)
Source: PLoS One. 2013 May 21;8(5):e63709. doi: 10.1371/journal.pone.0063709 (PMC3660571; doi:10.1371/journal.pone.0063709)
Supplement: Table S1 — Relationships between adaptation performance and clinical/demographic characteristics for subjects with Autism Spectrum Disorder (ASD). (DOC) [file pone.0063709.s001.doc]

Table S1. Relationships between adaptation performance and clinical/demographic characteristics for subjects with Autism Spectrum Disorder (ASD).

|  | Adapt. Rate | Amp. SD | Age | IQ | ADOS | ADI  Social | ADI Comm |
| --- | --- | --- | --- | --- | --- | --- | --- |
| Adapt. Rate | -- |  |  |  |  |  |  |
| Amp. SD | .57** | -- |  |  |  |  |  |
| Age | -.03 | -.07 | -- |  |  |  |  |
| IQ | -.14 | -.01 | .05 | -- |  |  |  |
| ADOS | .03 | .12 | .09 | -.07 | -- |  |  |
| ADI Social | -.02 | -.13 | -.01 | .01 | -.12 | -- |  |
| ADI Comm. | .15 | -.03 | -.12 | -.01 | -.11 | .68** | -- |

** p<.01

Adapt. Rate: rate of adaptation; Amp. SD: trial-to-trial variability of saccade amplitude during adaptation; IQ: full-scale IQ from the WASI; ADOS: social+communication total for the ADOS; ADI Social: social algorithm score from the ADI; ADI Comm.: communication algorithm score from the ADI.
