# Supplementary material for: Saccade Adaptation Abnormalities Implicate Dysfunction of Cerebellar-Dependent Learning Mechanisms in Autism Spectrum Disorders (ASD)
Source: PLoS One. 2013 May 21;8(5):e63709. doi: 10.1371/journal.pone.0063709 (PMC3660571; doi:10.1371/journal.pone.0063709)
Supplement: Table S2 — Relationships between adaptation performance and demographic characteristics for healthy control subjects. (DOC) [file pone.0063709.s002.doc]

Table S2. Relationships between adaptation performance and demographic characteristics for healthy control subjects.

|  | Adapt. Rate | Amp. SD | Age | IQ |
| --- | --- | --- | --- | --- |
| Adapt. Rate | -- |  |  |  |
| Amp. SD | .50** | -- |  |  |
| Age | .15 | .22 | -- |  |
| IQ | -.06 | -.11 | .02 | -- |

** p<.01

Adapt. Rate: rate of adaptation; Amp. SD: trial-to-trial variability of saccade amplitude during adaptation; IQ: Full-Scale IQ from the WASI;
