# Supplementary material for: Saccade Adaptation Abnormalities Implicate Dysfunction of Cerebellar-Dependent Learning Mechanisms in Autism Spectrum Disorders (ASD)
Source: PLoS One. 2013 May 21;8(5):e63709. doi: 10.1371/journal.pone.0063709 (PMC3660571; doi:10.1371/journal.pone.0063709)
Supplement: Table S3 — Saccade characteristics of individuals with autism spectrum disorders (ASD) and healthy controls during baseline testing, adaptation and recovery. (DOC) [file pone.0063709.s003.doc]

Table S3. Saccade characteristics of individuals with autism spectrum disorders (ASD) and healthy controls during baseline testing, adaptation and recovery.

|  | ASD | Controls | p |
| --- | --- | --- | --- |
| **Baseline** |  |  |  |
| Latency (ms) | 189 (33) | 187(27) | ns |
| Mean Velocity (°/s) | 366.09 (63.25) | 359.64 (44.39) | ns |
| Duration (ms) | 72.50 (12.96) | 71.20 (9.99) | ns |
|  |  |  |  |
| **Adaptation (rate of change^1^)** |  |  |  |
| Latency (ms) | β=.90 (SE=.69) | β=.89 (SE=.96) | ns |
| Mean Velocity (°/s) | β=-9.15 (SE=.87)* | β=-11.56 (SE=1.27)* | ns |
| Duration (ms) | β=-.01 (SE=.24) | β=-.22 (SE=.23) | ns |
|  |  |  |  |
| **Recovery (rate of change^1^)** |  |  |  |
| Latency (ms) | β=1.79 (SE=1.30) | β=-1.43 (SE=1.83) | ns |
| Mean Velocity (°/s) | β=-7.27 (SE=1.73)* | β=-3.45 (SE=.73)* | ns |
| Duration (ms) | β=.19 (SE=.05)* | β=.20 (SE=.07)* | ns |

^1^negative values indicate reduction over trials

* p<.001
